# New Insights into the Evolution of the Human Diet from Faecal Biomarker Analysis in Wild Chimpanzee and Gorilla Faeces

**DOI:** 10.1371/journal.pone.0128931

**Published:** 2015-06-10

**Authors:** Ainara Sistiaga, Richard Wrangham, Jessica M. Rothman, Roger E. Summons

**Affiliations:** 1 Massachusetts Institute of Technology, Cambridge, United States of America; 2 Universidad de La Laguna, La Laguna, Spain; 3 Harvard University, Cambridge, United States of America; 4 Hunter College CUNY, New York, United States of America; University of Florence, ITALY

## Abstract

Our understanding of early human diets is based on reconstructed biomechanics of hominin jaws, bone and teeth isotopic data, tooth wear patterns, lithic, taphonomic and zooarchaeological data, which do not provide information about the relative amounts of different types of foods that contributed most to early human diets. Faecal biomarkers are proving to be a valuable tool in identifying relative proportions of plant and animal tissues in Palaeolithic diets. A limiting factor in the application of the faecal biomarker approach is the striking absence of data related to the occurrence of faecal biomarkers in non-human primate faeces. In this study we explored the nature and proportions of sterols and stanols excreted by our closest living relatives. This investigation reports the first faecal biomarker data for wild chimpanzee (*Pan troglodytes*) and mountain gorilla (*Gorilla beringei*). Our results suggest that the chemometric analysis of faecal biomarkers is a useful tool for distinguishing between NHP and human faecal matter, and hence, it could provide information for palaeodietary research and early human diets.

## Introduction

The emergence of our genus has been often interpreted as a convergence of different anatomical, physiological and social changes attributed mainly to our tendency to eat more meat. The timing and the significance of this critical dietary adaptation and earliest hominin strategies for meat procurement have been the subject of vigorous debate [1–9].

Although there is a substantial amount of information available on the timing of butchery [10–14] and about the evolutionary benefits of eating meat [see for example, 15–17, 7], little is known about the proportions of animal protein intake necessary to influence hominin biology. In addition, there is potential confusion among the different interpretations of the role of plants in the crucial period in which the essential features of our physiology were shaped. Interpretations range from the important role of nutrient dense plants, such as nuts and seeds [18], and the role of tubers [19–21] to the meat-eating hypotheses that attributed the main role to the consumption of animal tissues [22–26].

Studies designed to elucidate aspects of predatory behaviour in non-human extant primates and modern hunter-gatherers have also shed some light into the importance of animal food consumption for hominoids [27–31]. For instance, some level of meat eating has characterized mostly all hominoids [18] but vertebrate hunting can be considered a synapomorphy of the sister taxa of panins (chimpanzees and bonobos] and hominins [3]. Substantial evidence shows that the ancestral lineage that led to humans had a plant-based diet [32–33]. Thus, an increase in animal tissue intake may have had significant evolutionary consequences some time after our divergence from an ape common ancestor. However, can the timing of this crucial dietary shift be identified more accurately? And, how much meat would make a difference?

So far, there is scarce evidence to suggest that the predatory behaviour of australopithecines differed from other apes [18]. Their carnivory probably was very similar to chimpanzees, resorting to occasional and opportunistic hunting to obtain a small percentage of their food [18, 34]. Nevertheless, the emergence of Pleistocene hominins encompassed the rise of traits such as increased body size, reduced gut size, higher brain capacity and extended life spans, all of which anthropologists have traditionally associated to a shift towards high quality food sources such as meat. Increased amounts of lipids and proteins are presumably necessary to make these changes possible.

Animal tissues are rich in proteins, micronutrients and lipids, even if in wild prey muscle tissues are likely more lean [22]. However, although protein quality if lower in plants, which are also normaly poor in lipids, apes can obtain substantial amounts of protein from plants; meeting or exceeding their estimated requirements, [34–43]. Given that lipids in plants are mostly found in fruits, their intake is dependent on the season [34].

Early human paleodietary reconstructions have undergone considerable advance with the incorporation of fossil bone isotopic analysis [29, 44–46]. However, carbon isotope analysis cannot readily distinguish among plant-based, meat based, and omnivorous diets [44–46]. Thus, our current view of early human diets, which is based on the fossil evidence of tooth wear patterns [47–49], associated tools [50–53], bone cut marks [54, 13] and isotopic bone data, does not provides information about the relative amounts of different types of food that contributed most. What were our ancestors’ regular meals? How much meat did they eat?

We previously reported [55] a successful approach to the Neanderthal diet using faecal biomarkers as direct indicators of the proportions of animal and plant intake. The use of biomarkers, more specifically faecal sterols (5β-stanols), has been widely employed to distinguish between different faecal pollution sources [56–60]. These lipids are known to be very stable, more resistant to degradation than other types of molecules such as DNA, proteins or carbohydrates and preserved in low thermal grade sediments through the Cenozoic [61–62]. The preservation of sterol molecules in Paleozoic sediments [63] suggests that geologic age is not a constraint for the application of this technique throughout the time span of human evolution. Moreover, lipids are not soluble in water and their decay is limited once they are incorporated into a sediment matrix [64].

Among the different types of lipids, we use 5β-stanols as biomarkers because such metabolic products are uniquely formed through microbial action of cholesterol and phytosterols in the gut of most higher mammals. The sterols used as dietary biomarkers are ubiquitously found among other mammals, and what makes them diagnostic is their relative distribution characteristic of their diet [55, 65, 62]. However, in some cases this distribution may be undiagnostic and the application of ratios [66–73] between stanols and sterols of animal and plant origin can be used to highlight the differences.

Coprostanol, a 5β-stanol, is formed through microbial saturation of its precursor Δ^5^-sterol cholesterol by specific bacteria present in the gut of higher mammals. Coprostanol results from converting both ingested and endogenously biosynthesized cholesterol. Humans cannot synthesize these and other stanols and thus they represent direct dietary input of plant sterols [55, 66, 73]. The combination of an animal’s diet, specifically the amount of plant material, the endogenous cholesterol biosynthesis and the efficiency of the gut microbial action, provide a distinct chemical signature that allows us the identification of the faecal origin and the dietary habits [56]. Thus, identification and quantification of 5β-stanols, particularly coprostanol, is an effective analytical strategy to approach the composition of human diets.

Conversion of cholesterol to coprostanol in humans is bimodal with a small percentage of people that are low to non-converters (less than one-third of coprostanol into the total faecal sterol content) and a majority of efficient converters. [74–75]. This microbial conversion starts in humans at the end of the first year and its efficiency is determined by the abundance of cholesterol-reducing bacteria [76–77]. An efficient conversion would decrease the risk of cardiovascular disease through the elimination of excess cholesterol from the body [78–79].

The first substantial meat eaters were presumably not prepared for a transition to a high fat and high cholesterol diet, even if intake of oily seeds and nuts prior to this dietary change would probably have favoured a better lipid digestion [29]. The shift from an herbivorous ape diet to a more omnivorous Pleistocene hominin diet required a fat and cholesterol-related adaptation that some studies have attributed to the acquisition of genes enabling high meat intake without risk of hypercholesterolemia [80–81]. Cholesterol increase from meat intake in early hominins should be indicated by an abrupt enrichment in coprostanol and an associated reduction in 5β-stigmastanol (a plant stanol) in their faeces. Based on our positive results with ca. 50 ka old Neanderthal samples and the preservation potential of sterols in ancient sedimentary environments, we suggest that this hypothesis is testable in Pleistocene hominin sites.

But first, due to the lack of faecal biomarker studies of wild NHPs it is necessary to undertake a preliminary analysis of the nature and proportions of sterols and stanols excreted by our closest living relatives. Until now, all available data on neutral lipid analysis of NHP faeces have been obtained from captive specimens [82–83], mainly Rhesus monkeys [84–85] and baboons [86], none of them hominines.

Vascular disease such as hypercholesterolemia is common among captive chimpanzees and gorillas when fed with commercial chow. Their nutrition in captivity often includes dairy products and animal fat as much as 5 to 10%, which notably deviates from the 2.5% fat intake in a wild ape diet [81]. To date, there is a lack of information about the prevalence of this pathology among wild communities.

Since the mechanism leading to these pathologies in great apes are also likely to have operated among early humans, wild NHP studies should be of value in understanding the mechanisms that regulated high fat and cholesterol intake in the earliest stages of human evolution. This paper reports differences in the faecal excretion of cholesterol and phytosterols as well as their degradation products in chimpanzee (*Pan troglodytes*) and mountain gorilla (*Gorilla beringei*) faeces.

## Materials and Methods

### Sample collection

10 fresh faecal samples of chimpanzees (*Pan troglodytes*) were collected from the Kanyawara community of Kibale National Park in Uganda in 2001. Faeces from known individuals were stored in individual vials with enough silica gel to keep them dry and then stored at room temperature. The Kanyawara chimpanzee community inhabits the north-western edge of Kibale National Park at around 1400 m amsl. The territory of the Kanyawara chimpanzees is a mix of mostly primary unlogged mid-altitude forest, with areas of partly logged forest, papyrus swamps and former pine plantations [87–88]. During a period of fifteen years, the community occupied 41 km^2^ with an annual median home range of 16.4 km^2^ [89]. Seven samples were from adult males; three (CH7, LR; CH1, AL; CH4, OT) were from adult females.

Additionally, 4 fresh faecal samples were collected from Mountain gorillas (*Gorilla beringei*) (2 silverbacks, 2 females) living in Bwindi Impenetrable National Park (BINP) in Uganda. The sampling was conducted in the Ruhija site of BINP during August 2002 to July 2003 in a group of gorillas that have been monitored daily since the mid-1990s. After being collected, all samples were air dried, milled to 1mm, transferred into individual vials and stored at room temperature [42].

### Ethics Statement

This work was in compliance with the laws of Uganda. The Uganda Wildlife Authority and The Uganda National Council for Science and Technology approved this research. Ethical clearance for this study was granted by the Harvard University’s IACUC with the protocol number 96–03 for non-invasive faecal collection. The Cornell University IACUC reviewed this work and decided that no protocol was necessary for non-invasive faecal collection. Researchers did not interact with primates at any point of this study.

### Sample preparation and solvent extraction

Subsamples of 0.5 to 1 g were collected for extraction. The samples were extracted ultrasonically 3 times, 30 minutes each with 3:1 v/v CH_2_Cl_2_ (DCM): MeOH (methanol) to obtain the total lipid extract (TLE). Solvent was removed from the TLEs under a constant low-velocity stream of N_2_ gas using a TurboVAP, and dried samples were stored under nitrogen in glass vials with Teflon-lined caps. The TLEs were saponified by the addition of 5 mL of 2 N sodium hydroxide in 90% methanol to the TLE by heating at 100°C for 1 hour. After being cooled to room temperature, sterols were extracted in hexane, and the solvent was evaporated to dryness under a gentle stream of nitrogen at 60°C. After extraction of sterols, 10 mL of distilled water was added to the aqueous solution, which was then acidified to pH 3–4 with 2 N HCl, and the acid fraction was recovered in ethyl acetate. The solvent was removed under a gentle stream of nitrogen at 60°C. Both residues were derivatized by addition of 50 μg of pyridine and 50 μg of BSTFA containing 1% of TMS and heated to 60°C for 30 minutes. Samples were then diluted with an appropriate volume of hexane prior to GC-MS analysis.

### GC-MS Analyses

The analyses were carried out using full scan mode, with the following parameters: 5 μL injected via automatic liquid sampler; the oven was temperature programmed with an initial isothermal of 2 min at 40°C followed by an increase to 350°C at 10°C per minute followed by a final isothermal at this temperature of 15 min. Helium was used as carrier gas and held at a constant flow at 2.0 ml per minute throughout the analysis. Identification was performed using a Hewlett Packard 6890 series gas chromatograph–mass spectrometer equipped with a 5% phenyl methyl siloxane column (HP-5, 60m, 0.25mm i.d., film thickness 0.25 μm) Compound identification was achieved by interpretation of characteristic mass spectra fragmentation patterns, gas chromatographic relative retention times, and by comparison with literature.

### Statistical analysis

Statistical analysis was carried out using the SPSS software version 19 and the R freeware (www.r-project.org). All data were assessed for normality and transformed as appropriate. We have made a cluster analysis followed by a discriminant analysis with the raw data to show major trends and groups. The cluster analysis was made using the average linkage clustering method (UPGMA). We also conducted bivariated correlation analysis using Microsoft excel 2010. Additional calculations were performed using standard analyses programmed in the SPSS software. A Multivariate Analysis of Variance (MANOVA) analysis was made using R, using the "Hotelling-Lawley" test, to compare the joint variance difference of the three groups (chimpanzees, gorillas and neanderthals) regarding the different sterol compounds. When significant differences were documented, a pairwise comparison was carried out combining an R "manova" function which enabled the creation of subsets of pair groups and the "summary.aov" function which tested their significance. Significant differences (with alpha values under 0.05) showed that the lipid composition of the paired group compared was different.

## Results

### Non-human primate faecal biomarker analysis

**Fig 1:**
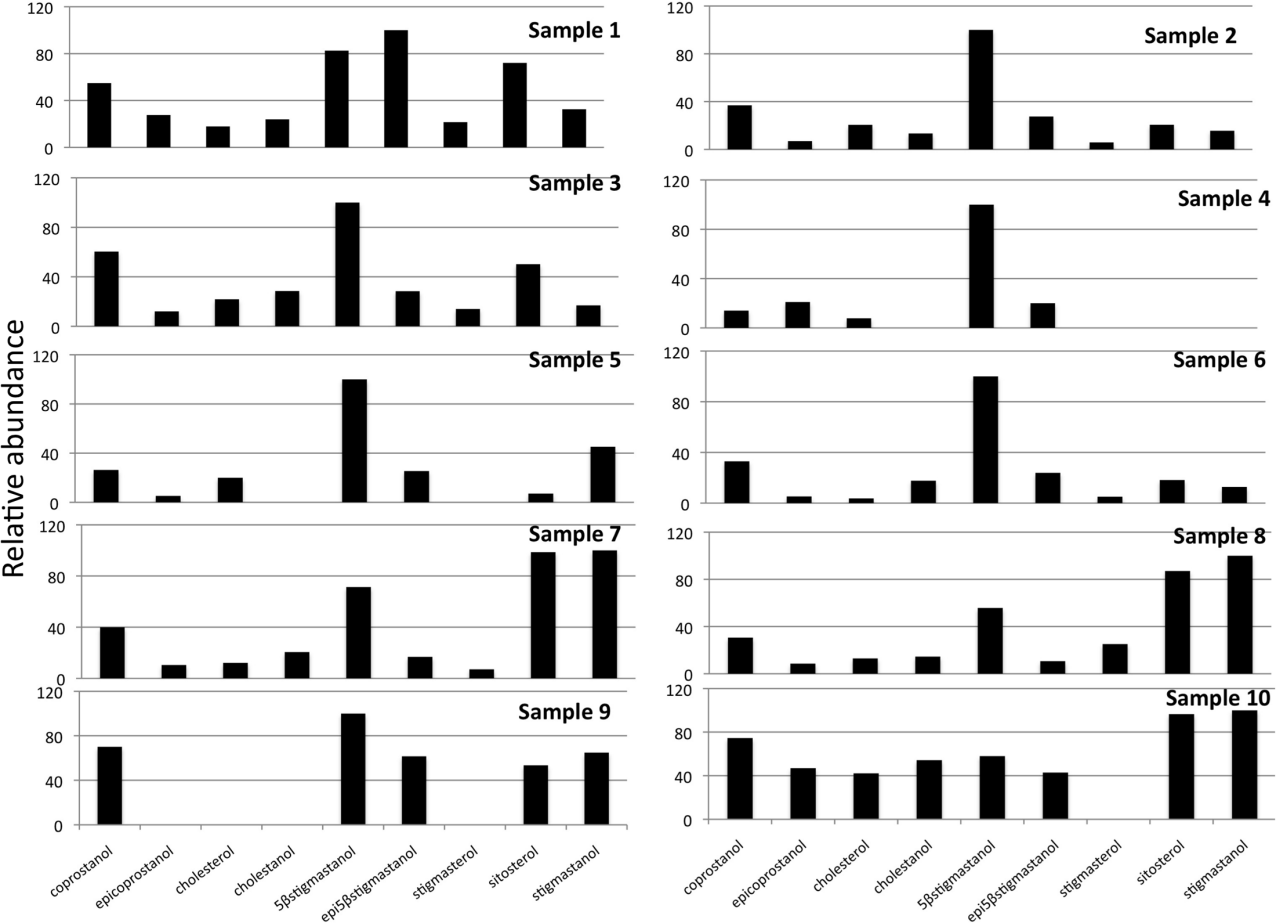
Sterol fraction histogram from the chimpanzee samples. Relative concentrations are expressed as % of the largest peak compared to TIC (total ion chromatogram) in the chromatogram.

**Fig 2:**
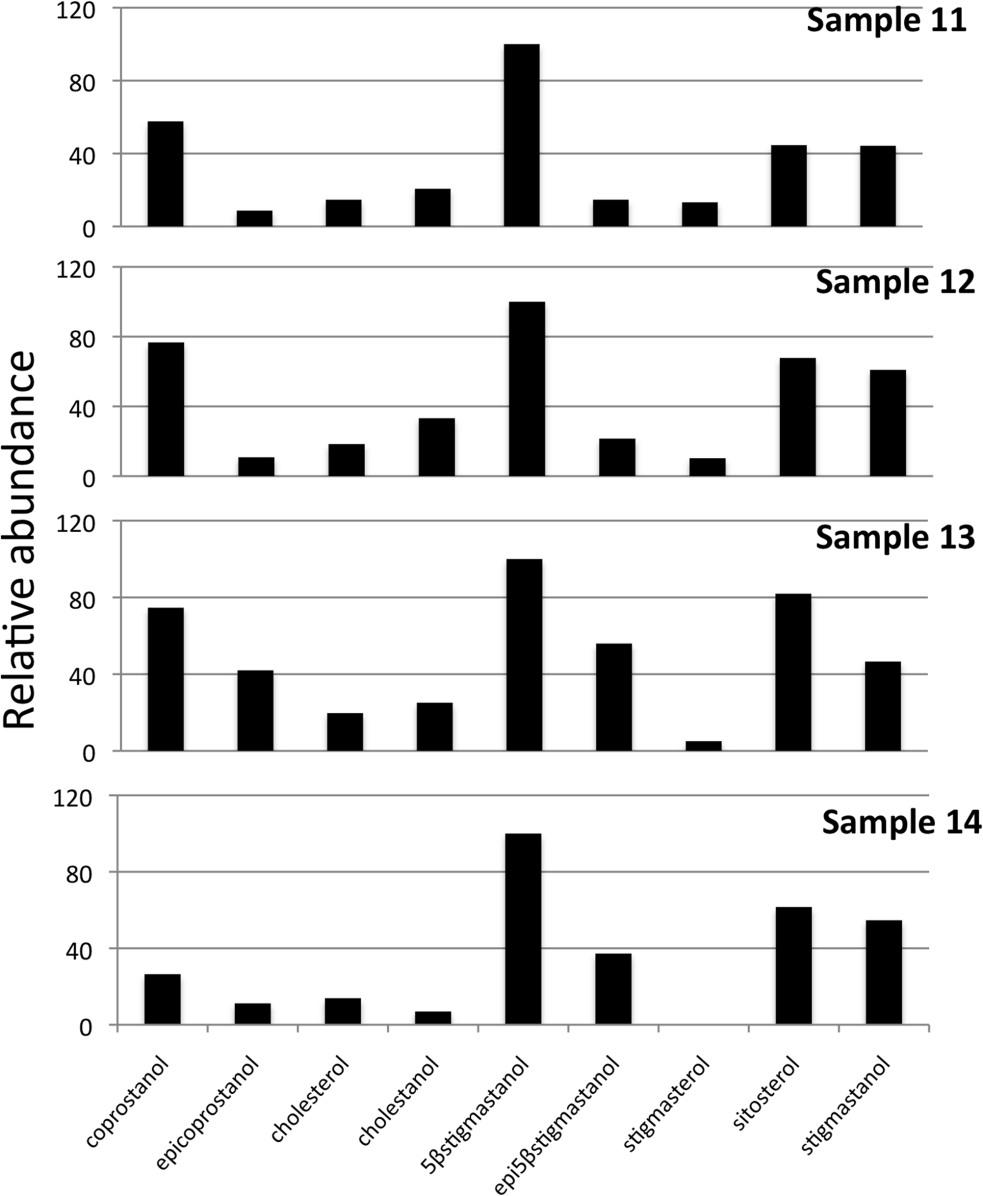
Sterol fraction histogram from the gorilla samples. Relative concentrations are expressed as % of the largest peak compared to TIC in the chromatogram.

The results of faecal sterol composition in gorillas and chimpanzees are shown in Table 1 and Figs 1 and 2. Although their sterol excretion patterns seem very similar, some differences exist. In both species, plant sterols represent the major portion of the neutral steroids present in the faeces. However, the main differences are observable in the amount of animal source sterols (coprostanol, epicoprostanol, cholesterol and cholestanol) (Table 1). Coprostanol content is significantly higher in gorilla females (samples 12 and 13) and in chimpanzee sample 10. Interestingly, sample 10 (KK-CH3) was collected after the subject (adult male Kakama) had been 3 days outside the observation area. The same subject was sampled (KK-CH5) a few days later, inside the observed area. This second sample yielded a sterol pattern similar to the rest of the chimpanzee samples: predominant occurrence of 5β-stigmastanol with significant amounts of phytosterols and a very low animal sterol content.

**Table 1:** Samples and relative abundance of sterol content.

Sample	Code	Nature	Sex	coprostanol	epicoprostanol	cholesterol	cholestanol	5β-stigmastanol	epi5β-stigmastanol	Sitosterol	stigmastanol	Stigmasterol
1	CH7, LR	Chimpanzee	Female	54,85	27,64	17,87	23,95	82,58	100	72,1	32,6	21,53
2	CH8,PG	Chimpanzee	Male	36,9	6,97	20,56	13,34	100	27,51	20,61	15,64	5,91
3	CH5,KK	Chimpanzee	Male	60,44	12,05	21,84	28,51	100	28,36	50,14	16,93	13,98
4	CH6,ST	Chimpanzee	Male	14,03	21	7,75	0	100	20,02	28,96	7,36	8,48
5	CH8,PG	Chimpanzee	Male	26,34	5,21	19,96	0	100	25,37	7,08	45,22	0
6	CH9,LK	Chimpanzee	Male	32,96	5,29	3,7	17,7	100	23,87	18,23	12,76	5,07
7	CH4,OT	Chimpanzee	Female	39,9	10,44	12,11	20,55	71,34	16,75	98,62	100	7,06
8	CH1,AL	Chimpanzee	Female	30,62	8,59	12,97	14,53	55,75	10,65	87,01	100	25,15
9	CH2,YB	Chimpanzee	Male	70,06	0	0	0	100	61,52	53,47	64,88	0
10	CH3,KK	Chimpanzee	Male	74,49	46,83	42,12	54,21	57,87	42,82	96,5	100	0
11	G3,SB2 ZS	Gorilla	Male	57,65	8,67	14,65	20,65	100	14,66	44,59	44,25	13,22
12	G4,AF2 MT	Gorilla	Female	76,65	10,81	18,4	33,21	100	21,51	67,76	60,95	10,26
13	G2,AF1 BN	Gorilla	Female	74,63	41,97	19,67	25,13	100	55,94	81,93	46,57	5,08
14	G1, SB1 KK	Gorilla	Male	26,46	11,18	13,88	6,91	100	37,24	61,59	54,66	0
15	NEAN. H32 SN	Neanderthal		51,38	100	10,77	11,67	1,1	0,47	3,26	2,06	1
16	NEAN. H32 GL	Neanderthal		33,41	100	22,94	18,36	5,12	3	23,29	14,37	57,71
17	NEAN. NIX	Neanderthal		100	0	21,71	20,31	0	0	37,65	7,73	68,72
18	NEAN. NIX	Neanderthal		100	0	6,82	0	0	0	0	0	0
19	NEAN. H44 WL	Neanderthal		85,58	33,07	100	30,52	39,44	27,05	28,54	24,107	0

Table 1 shows the samples collected, their number, code and nature. Also, we present their relative neutral sterols content. Values are standardized to the highest peak in the chromatogram. Relative concentrations are expressed as % of the largest peak compared to TIC (total ion chromatogram) in the chromatogram.

When applying the ratio (Table 2) proposed by Bull and co-workers [65] to differentiate between herbivore and human/pig faecal matter, we observe that except for sample 10, all the faecal samples collected from chimpanzees are in agreement with values expected for a mammal whose diet is composed predominantly by plant food.

**Table 2:** Stanol and sterol ratios.

Sample number	1	2	3	4	5	6	7	8	9	10	11	12	13	14	15	16	17	18	19
ratio stanol C27/C29	0,45	0,34	0,54	0,29	0,25	0,3	0,57	0,59	0,43	1,2	0,57	0,71	0,74	0,27	96,42	16,42			1,78
ratio sterol C27/C29	0,39	0,93	0,75	0,21	0,38	0,69	0,16	0,14	0	0,49	0,39	0,4	0,34	0,17	4,21	1,09	0,92	6,82	2,47

Table 2 shows the values obtained when applying the ratio proposed by Bull and co-workers [65]. Values below 1 have been used as diagnostic for an herbivore diet, whereas values higher than 1 are characteristic of humans. The second ratio is related to sterols of animal origin (C27) and plant origin (C29).

Preliminary results from mountain gorilla faeces show two different patterns related to coprostanol content (Fig 2). Silverbacks (sample 7 and 11) present a sterol profile dominated by 5β-stigmastanol with a low occurrence of coprostanol and sterols of animal origin. In females (samples 12 and 13), although 5β-stigmastanol is also dominant, there is a significant amount of coprostanol, in levels that far exceed those expected for a complete herbivore.

A MANOVA test yielded significant differences in lipid composition among the three groups (chimpanzees, gorillas and neanderthals): Wilks (p = 2.2e-16) and Hotelling-Lawley (p = 2.2e-16) tests yielded the same significant result. The pairwise comparison showed that the differences were between the neanderthal sample and the gorilla (p = 0.004) and chimpanzee (p = 0.010) samples. Gorillas and chimpanzees were very similar when comparing all lipid types together (p = 0.91). Significant differences in 5β-stigmastanol (p = 0.008), epi5β-stigmastanol (p = 0.04), sitosterol (p = 0.025) and stigmastanol (p = 0.023) were found when comparing the three groups.

**Fig 3:**
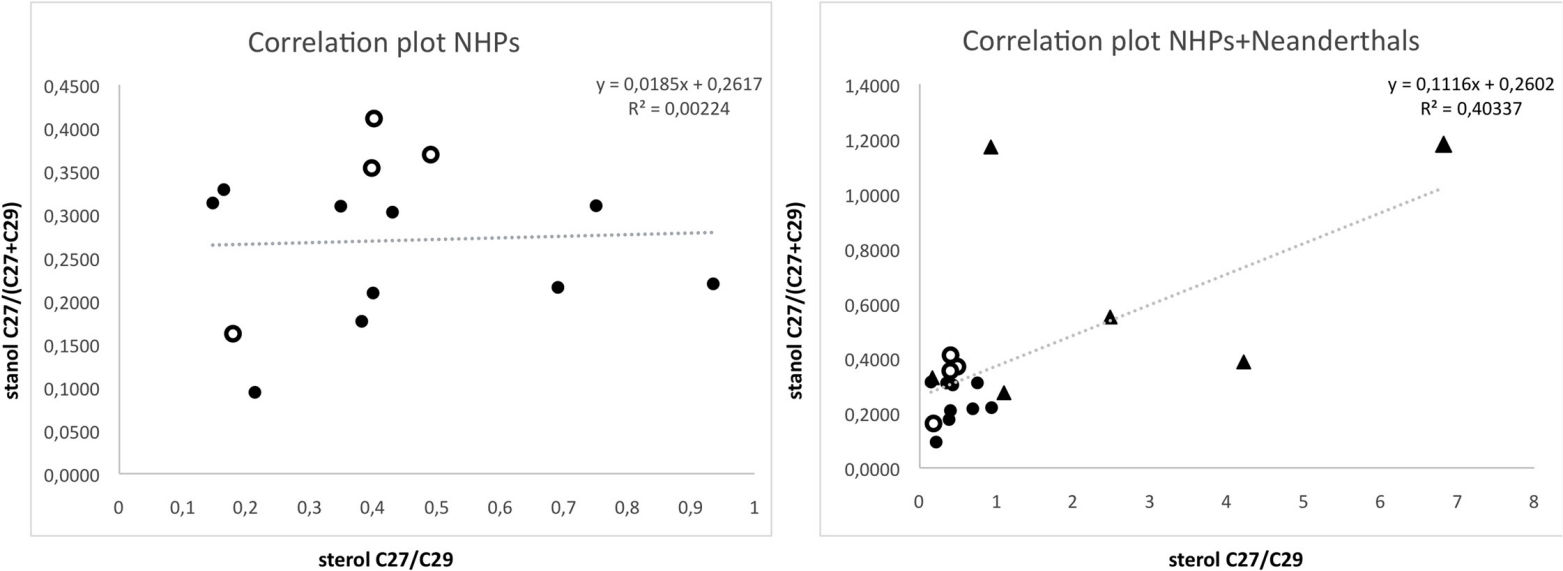
Correlation plot. Two correlation plots are presented. The first shows the correlation between the 5β-stanol ratio C27/(C27+C29) (Y axis) and the sterol ratio C27/C29 (X axis) in the NHP samples. Given that C29 stanols were not present in some samples we have applied the 5β-stanol ratio C27/(C27+C29) instead of the ratio stanol C27/C29 [65]. Gorillas are shown as white circles. The second plot shows the same correlation but including Neanderthal samples (triangles).

Fig 3 shows a two correlation plot comparing the ratio between 5β-stanols with 27 carbons (animal origin) and 5β-stanols with 27 carbons + 29 carbons (plant origin) in the y axis; and the ratio between sterols composed by 27 carbons (animal origin) and 29 carbons (plant origin) in the x axis. In the first plot, we observe a lack of correlation in NHP samples, which are more or less grouped together except for sample 10. It must be noted that it is not possible to distinguish between chimpanzee and gorilla samples. However, when we include the values obtained in our previous study with Neanderthal samples, there is a clear separation of the NHPs on the one hand, clustered, and the Neanderthals on the other, which show a significant inter-individual variation.

### Discriminant Analysis

A canonical plot of the cases based on discriminant analysis was performed on the percentage relative abundances of Neanderthal faecal sterol profiles [55], Mountain gorilla and chimpanzee. We decide to include data from samples analyzed in our previous work on Neanderthals [55] to explore the possibility of overlapping between humans and our closest relatives.

**Fig 4:**
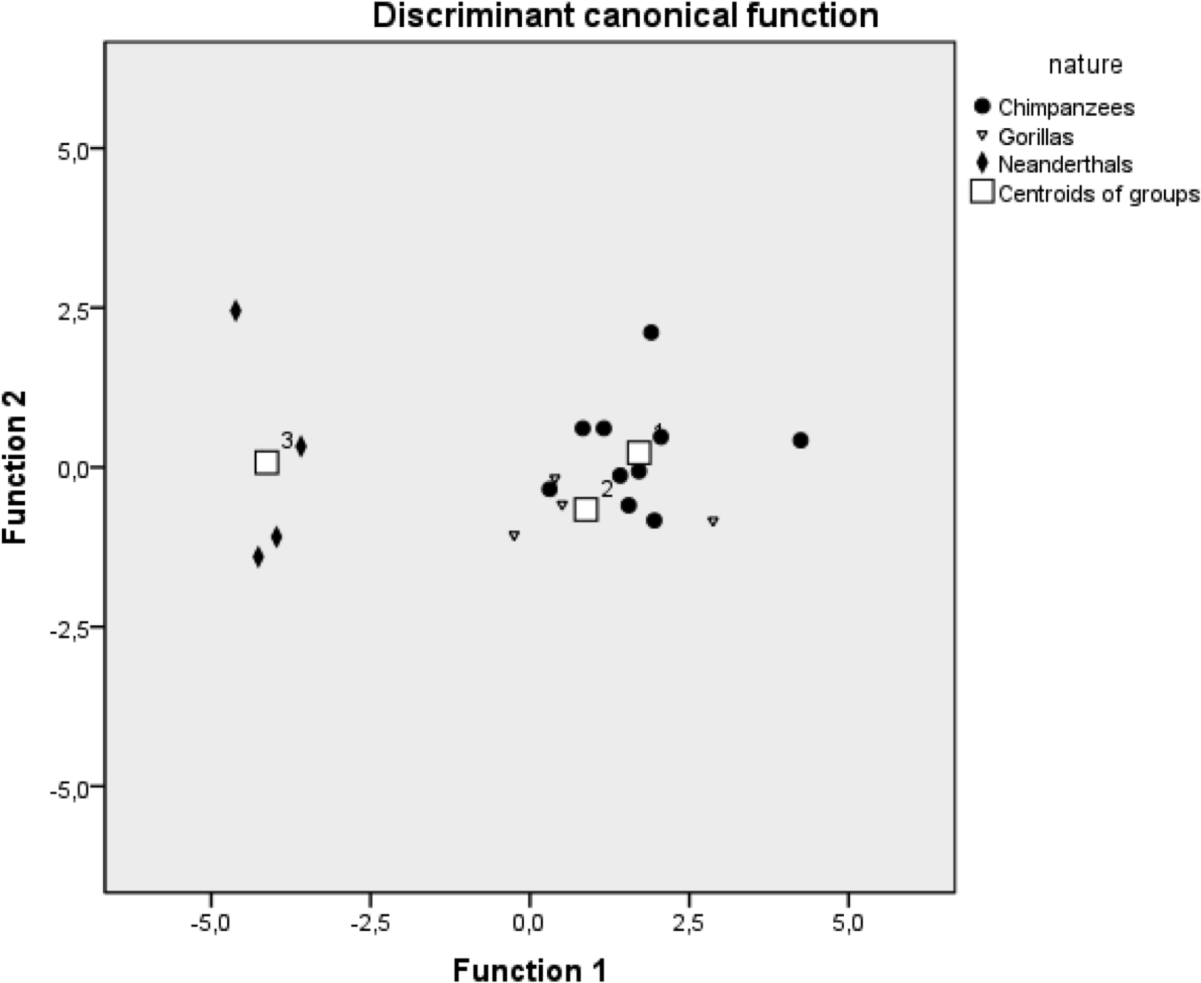
Discriminant analysis. The canonical discriminant analysis plot shows NHP and Neanderthal samples. Group 1 corresponds to the chimpanzee samples, the second to gorillas and the third to Neanderthals.

The canonical separation of cases is presented in Fig 4, showing two clear groups. One corresponds to Neanderthals, and the other is composed by chimpanzees and gorillas.

### Cluster analysis

**Fig 5:**
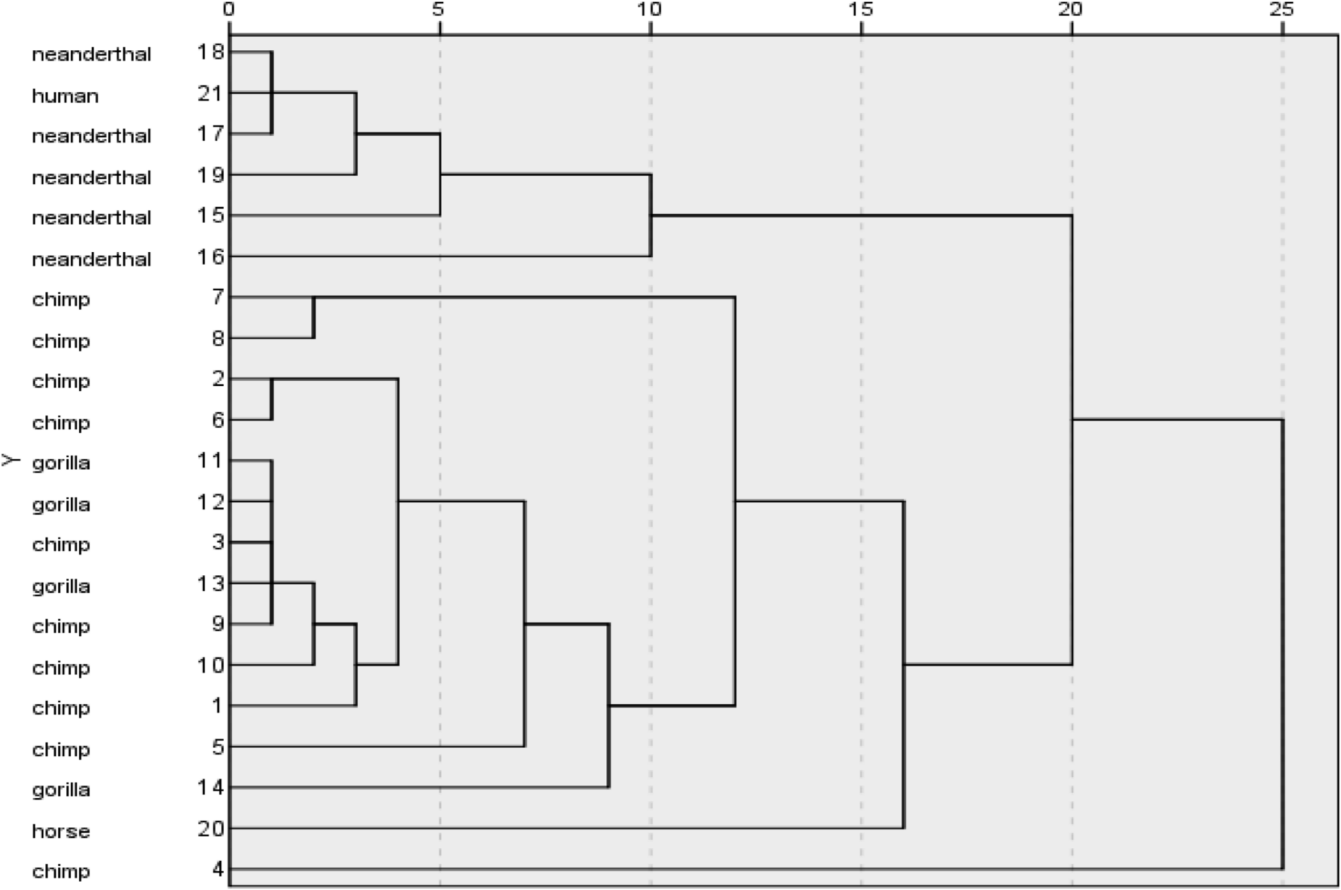
Cluster analysis. The dendrogram generated using the intragroup’s method of linkage. It shows groups formed when clustering the NHP samples with 5 neanderthal samples^55^ and 1 reference for modern human^56^.

Cluster analysis was used to assess the links between human species, gorillas and chimpanzees. We decided to include the data from a living human and a horse case reported in the work conducted by Leeming and colleagues [56]. We also tried to include dog and duck data from the same work but the orders of magnitude difference were too high to assess any interpretation. The dendrogram obtained when clustering samples is presented in Fig 5. The intragroup’s method of linkage provided a clear interpretation of the dendrogram, while other methods did not generate clear groups. This analysis allowed the establishment of two main groups with 4 orders of magnitude difference. This figure shows two clusters composed of 1 living human sample [56] and 5 Neanderthal samples [55] arranged in the first cluster; followed by a second cluster that includes gorilla and chimpanzee samples and one horse [56]; and a third cluster with an outliner chimpanzee, sample 4, separated by 5 orders of magnitude.

## Discussion

Taken together, our preliminary results show that the chemometric approach is a useful tool in the distinction between NHP and human faecal matter, and hence it could provide a new source of information for the study of the early human diet.

Faecal biomarkers have been demonstrated to be very useful in the characterization of the origin of the archaeological faecal deposits [e.g. 55,65,76,90–91]. Our approach enables us to accurately differentiate between human and non-human primates, while in some cases, such as chimpanzee sample 10, when applying the C27 stanol/C29 stanol ratio [65] this divergence was unidentifiable. It is also noteworthy that gorillas and chimpanzees, which are primates species with dissimilar types of diet, cannot be clearly differentiated neither using the ratio, nor through SDA.

While the primary aim of the study was to confirm the usefulness of our approach to the study of early hominin diet, the opportunity was also taken to record new data about the metabolism of fats and cholesterol in our closest relatives. We observed that sterol conversion seems to be high in both primates, Kibale chimpanzees and Bwindi gorillas. However, samples 7, 8 and 10 show a higher presence of sterol precursors (in this case, sitosterol and stigmastanol), indicating a low conversion sterol to 5β-stanol, whereas gorilla conversion seems to be much more efficient than chimpanzee’s. This could be explained by the colonic microbiome and long food-fermentation times that could enhance bacterial conversion in gorillas.

The group of NHPs displayed a wide inter-individual variability, but in all of the cases (except for sample 10) 5β-stigmastanol was the major sterol in their faeces and the phytosterols contributed more than 55% of the total sterols, which is consistent with previous findings [82–86]. By reviewing the data for the NHPs, it is clear that there is an evident pattern in the sterol profile (except for sample 10) corresponding to a folivorous and frugivorous diet. The main differences among subjects are related to the cholesterol and metabolites content in their faeces.

**Fig 6:**
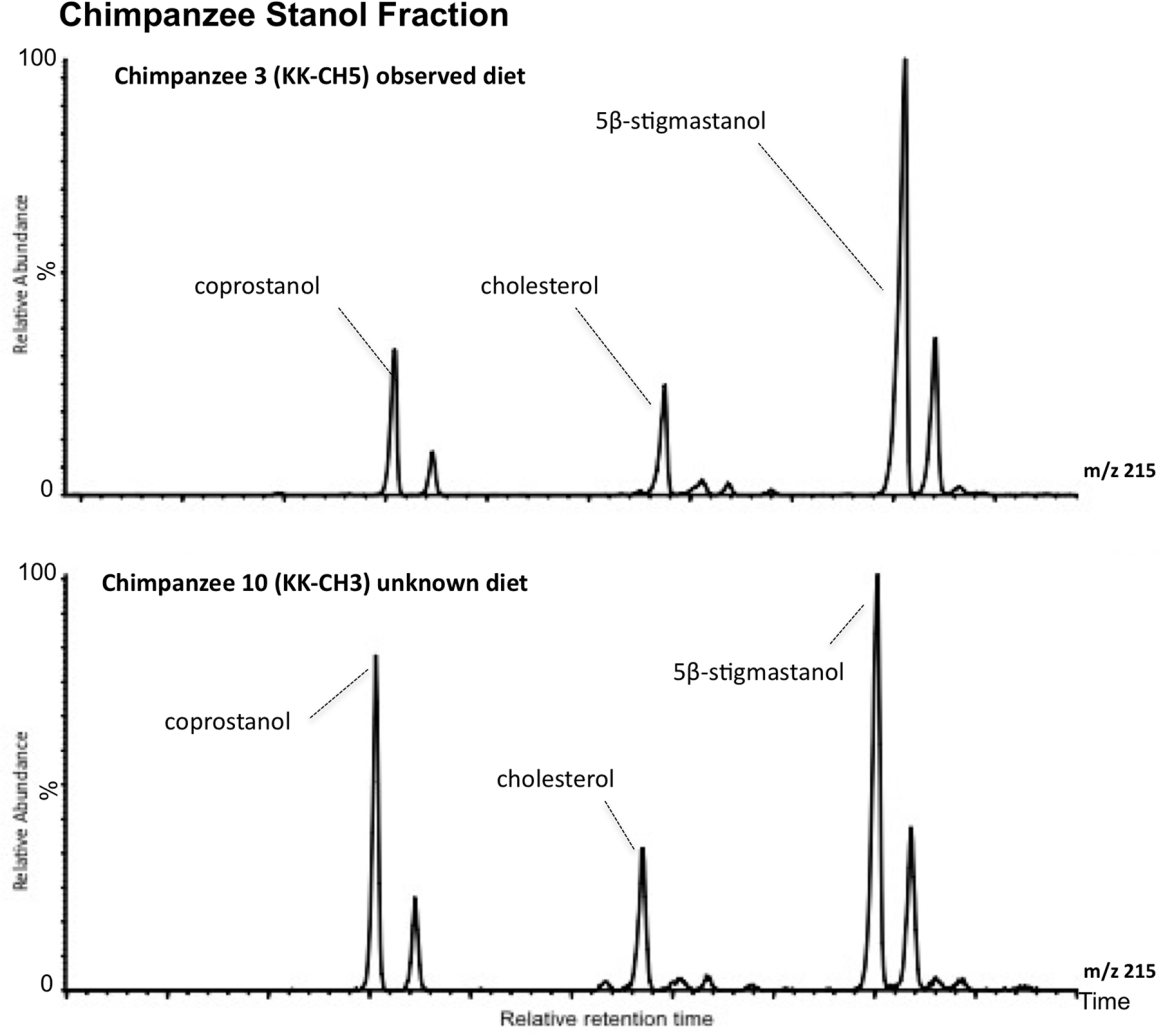
Chimpanzee stanol partial chromatogram. Comparison of the m/z 215 ion chromatograms for the neutral lipid fraction from sample 3 (CH5) and 10 (CH3) collected from the same chimpanzee in different moments.

Chimpanzees showed the widest distribution covering the ranges of coprostanol and cholesterol content characterized by humans, being remarkable in sample 10. When compared with sample 3 (Fig 6) (same subject different sampling time) sample 10 shows a striking increase in the cholesterol group, which occurred when this chimpanzee was not observed for three days. Given the levels of coprostanol observed, this was probably due to the ingestion of animal tissue. Meat intake in common chimpanzees has been estimated around 5% [30] and up to 10% in some individuals.

Given the levels of coprostanol observed, we suggest that this was due to the ingestion of animal tissue. Meat intake in common chimpanzees has been estimated to be around 5% of the diet in some populations and up to 10% in some individuals [30]. However the Kanyawara chimpanzees are not frequent hunters. Their diet includes ~75% ripe fruits and ~20% leaves and piths, all of which supply energy in the form of carbohydrates and protein rather than lipids [36,85, 91–92]. Most of the remaining ~5% of the diet is equally lipid-free (e.g. bark, wood, flowers). Insect-eating by Kanyawara chimpanzees is extremely rare (mostly small nests of wasps eaten a few times per year). The average chimpanzee in Kanyawara probably eats meat only about once per month, given that Wrangham and colleagues [92] found remains of vertebrates in 2.95 of chimpanzee faeces. However when they eat meat they consume enough to be consistent with the levels of coprostanol and cholesterol observed in sample 10.

**Fig 7:**
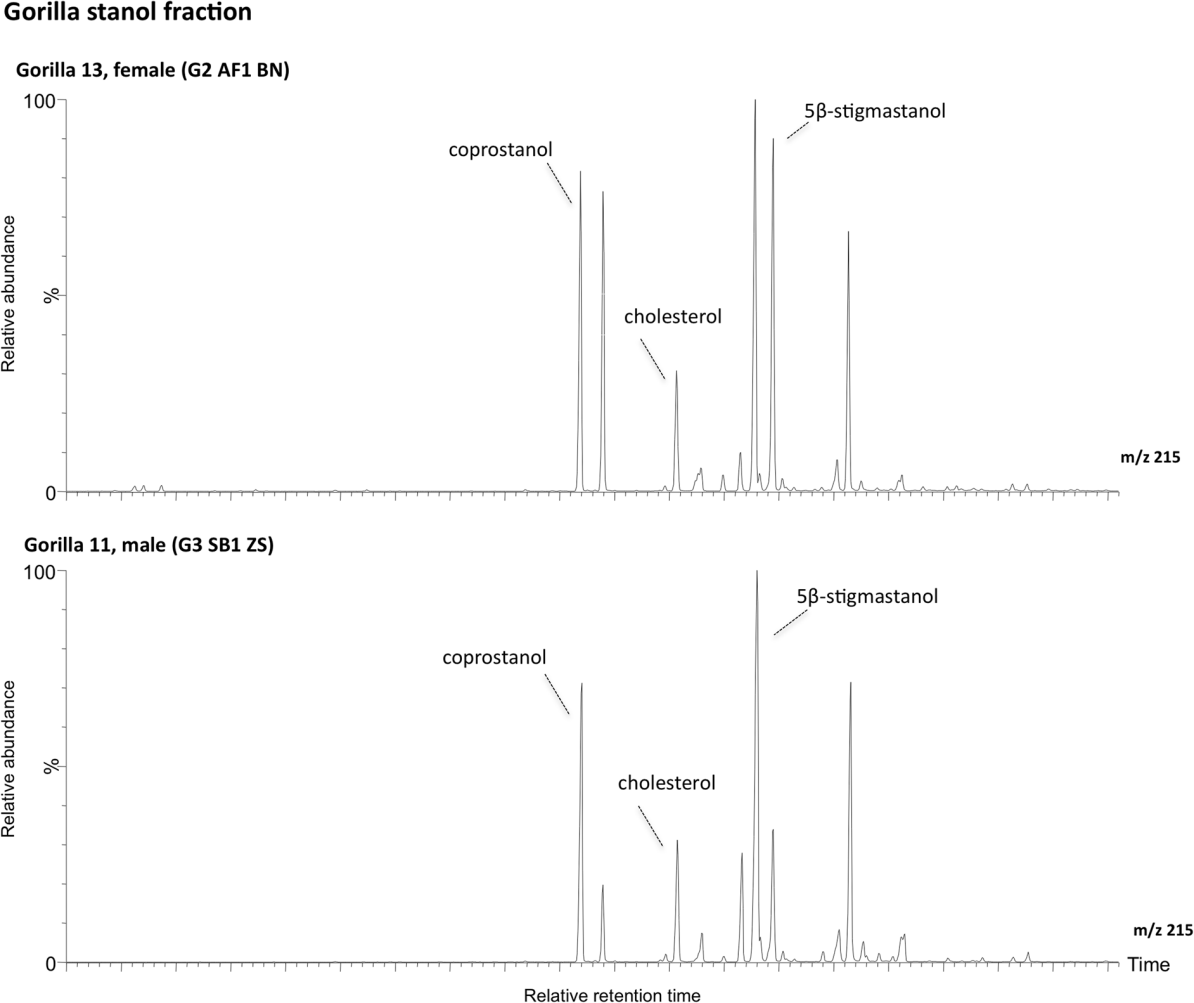
Gorilla stanol partial chromatogram. Comparison of the m/z 215 ion chromatograms for the neutral lipid fraction from sample 13 (Gorilla female) and 11 (Gorilla silverback).

Our data in Bwindi gorillas show a sex difference in coprostanol content (Fig 7) even if coprostanol and cholesterol amounts are higher in all the samples than those expected for a supposed complete herbivore. Mountain gorillas from BINP have a diet low in lipids [39] composed almost entirely of plant tissues that can include rarely ants or other insects [42]. Insects contain approximately 0,1% cholesterol (1mg/g tissue) like other animals, depending on species and diet [93–94]. It is possible that the accidental consumption of insects with vegetation affected these results.

On the one hand, due to the nutritional cost of reproduction and growth in gorillas, growing juveniles and adult females eat more food and thus more protein and fat per kg of metabolic body mass than silverbacks [42] in the form of a plant-based diet, and generally dietary fat represents a very small portion of the intake of gorillas [39]. Thus, it can be argued that this sexual difference in coprostanol excretion might be due to a difference in feeding behaviour. However, this is currently speculative and further analyses on a larger sample set are needed. Additional information about the cholesterol intake of healthy primate populations in the wild, however, is still lacking and should be addressed in the future.

BINP mountain gorillas are herbivorous and the greater content in coprostanol and cholesterol in their faeces is not consistent with a folivorous-frugivorous diet and may not be diet induced. Gorillas have large bodies, large colons, but proportionally short small intestines [95–96]. Ponz de Leon and colleagues [97] suggested that the transit time of small intestine might be a key factor in cholesterol absorption, accelerating cholesterol transit through the duodenum and jejunum where the bulk of the absorption takes place. A hypothesis for this greater concentration can be related to the higher content of phytosterols competing into the cholesterol pool, which may have interfered with cholesterol absorption, therefore increasing the excretion in their faeces. Gomez Zapata pointed out that microbiome may also have played an essential role in the increasing capacity for energy harvest and enhanced lipid metabolism in the energetic budget of Gorillas [98], which in turn might be also related to the coprostanol values observed in our samples. His results show differences in gorillas on a leaf-based diet and those that eat more fruit. The latter seem to have an increased cholesterol metabolism. This could be the case of our female mountain gorillas with an increased dietary breadth.

Neutral lipids excretion studied in cow faeces have shown a similar pattern not consistent with a complete herbivorous diet [56]. Unlike cholesterol, phytosterols are not subject to significant metabolic action and they are poorly absorbed [99]. Still they can compete with cholesterol for entry into micelles during digestion therefore reducing cholesterol absorption [84, 100–102].

A diet low in fat and cholesterol, yet accompanied by high coprostanol output, has also been reported in Taharumara Indians and interpreted as reflecting maximum cholesterol synthesis, stimulated by life-long low cholesterol, low fat diets [102–103]. The reason for this is not known, and the lesser amount of exogenous cholesterol entering to the body did not appear to entail a parallel marked shift in the amounts of cholesterol and metabolites in their faeces, indicating that cholesterol synthesis remains responsive to changes in dietary intake [103].

Several studies have documented hypercholesterolemia as a common pathology among captive gorillas [104–105] and chimpanzees [106–108]. Finch and Stanford [81] suggested that chimpanzees and gorillas, relative to other Old World primates, might be more sensitive to induced cholesterolemia, being five-fold more sensitive to dietary cholesterol than humans [109]. Although there is not much information about vascular diseases such as hypercholesterolemia in wild populations, this evident sensitivity to fat and cholesterol intake, if shared in a common ancestor, required adaptations that allowed the first persistent meat eaters around 2,5 ma to have a diet rich in fatty acids and cholesterol [80–81]. The health and life span of the earliest persistent meat eaters were probably affected by the negative effects of cholesterol intake.

Besides the impact of meat eating in cholesterol blood levels, animal tissues contain levels of nitrogen that are toxic for the foregut bacterial colonies when consumed in quantities as observed in humans [23, 110] and volumes of fibre that are too low to prevent colonic twisting in hindgut fermenters like gorillas or chimpanzees [29].

How then, did the hominin digestive system overcome the negative effects of meat-eating? A primitive gut with large colonic bacterial colonies would not have tolerated a shift from regular fruit and leaf eating to regular fat and meat eating [29]. Small increases in nuts and seeds first, and then animal tissues, probably allowed lipid digestion in the small intestine and a reduction in the size of the colon [29]. There might also have been a genetic mutation. Some studies have pointed to de acquisition of genes (ApoE alleles) allowing fat consumption without hypercholesterolemia [80–81]. This would have favoured the intake of PUFAs (polyunsaturated fatty acids), the necessary substrate for the formation of brain tissue [111 –112].

Access to preformed PUFAs omega 3 (with 6 double bonds) and omega 6 (with 4 double bonds) are exclusive to animal tissue-eaters since these fatty acids (FA) are not present or present in small amounts in plants [23, 79]. More specifically, animal fat provides a source of preformed long chain PUFAs, including arachidonic acid AA (22.4 n6) docosopentanoic acid DTA (22.5 n6) and docosohexanoic acid DHA (22.6 n3) which make up over the 90% of the long chain PUFAs found in brain grey matter of all mammals [113].

These FA have been considered a prerequisite for encephalization [23, 26, 114–116] and essential for prenatal and postnatal brain development [116–118] and retinal function [119–120]. However, Carlson and Kingston [121] argued that while conversion to DHA from alpha linoleic acid (ALA) remains insufficient in adult males (0 to 4%), conversion in women of reproductive age could reach 9%, increasing during the sixth week of gestation, hence providing enough DHA for normal foetal brain development.

Nevertheless, the disproportionately large human brain suggests a considerable intake of the nutrients required for a sustained trend of increasing encephalization [118]. These nutrients probably needed to be supplied in larger amounts than those provided by the mother for normal prenatal and postnatal brain development. Taking into account the energetically costly expansion of human brains [15] and the pumping role that these brain selective nutrients probably played in our evolution, we assume that food sources meeting both requirements (increased dietary quality and long chain PUFAs) were present in the original baseline diet prior to encephalization and following brain expansion of Pleistocene hominins.

Taking a look into the food sources available to early humans, no single food simultaneously fulfils both requirements [23]. The richest dietary source of preformed AA and DHA regularly available to hominins through hunting or scavenging was possibly ruminant brain that complemented with bone marrow possibly satisfied both requirements [23]. Evidence for fish consumption in early humans sites [122–126] suggests that, fish became a food source rich in brain selective nutrients around 2 Ma [119, 123]. Aquatic food sources as well as ruminant brain would supply amounts of DHA that far exceed the daily intake recommended (100mg) for a normal brain development in modern humans [23].

Indirect evidence of brain tissue consumption reflected in the remains of fresh broken skulls has been identified in early human sites [24, 127–128]. Ruminant brain and marrow contains around 2037mg /100 g [129] and 119-6mg/ 100g [130] cholesterol respectively. We believe, that such amounts would entail a significant increase in cholesterol and metabolites in early human sterol output, reflecting dietary changes.

The identification of the direct sources of these faecal sterols are still a challenge to overcome and we recognize the great complexity of determining exactly how each of these heterogeneous sources fits into the final pattern of steroid faecal excretion of early humans. In light of this, we believe that this study sets the stage for a future incorporation of faecal biomarkers into a holistic palaeodietary approach.
